# miRNA-132/212 Gene-Deletion Aggravates the Effect of Oxygen-Glucose Deprivation on Synaptic Functions in the Female Mouse Hippocampus

**DOI:** 10.3390/cells10071709

**Published:** 2021-07-06

**Authors:** Daniel Bormann, Tamara Stojanovic, Ana Cicvaric, Gabor J. Schuld, Maureen Cabatic, Hendrik Jan Ankersmit, Francisco J. Monje

**Affiliations:** 1 Center for Physiology and Pharmacology, Department of Neurophysiology and Neuropharmacology, Medical University of Vienna, Schwarzspanierstraße 17, 1090 Vienna, Austria; n1118880@students.meduniwien.ac.at (D.B.); tamara.stojanovic@meduniwien.ac.at (T.S.); n01629958@students.meduniwien.ac.at (G.J.S.); maureen.cabatic@meduniwien.ac.at (M.C.); 2 Laboratory for Cardiac and Thoracic Diagnosis, Department of Surgery, Regeneration and Applied Immunology, Medical University of Vienna, Research Laboratories Vienna General Hospital, Waehringer Guertel 18-20, 1090 Vienna, Austria; hendrik.ankersmit@meduniwien.ac.at; 3 Division of Thoracic Surgery, Medical University of Vienna, Waehringer Guertel 18-20, 1090 Vienna, Austria; 4 Department of Psychiatry and Behavioral Sciences, Albert Einstein College of Medicine, New York, NY 10461, USA; ana.cicvaric@einsteinmed.org; 5 Aposcience AG, Dresdner Straße 87/A 21, 1200 Vienna, Austria

**Keywords:** miRNA 132/212, oxygen-glucose deprivation (OGD), hippocampus, dentate gyrus, field excitatory postsynaptic potentials (fEPSP), ischemia, hypoxia, acetylcholine

## Abstract

Cerebral ischemia and its sequelae, which include memory impairment, constitute a leading cause of disability worldwide. Micro-RNAs (miRNA) are evolutionarily conserved short-length/noncoding RNA molecules recently implicated in adaptive/maladaptive neuronal responses to ischemia. Previous research independently implicated the miRNA-132/212 cluster in cholinergic signaling and synaptic transmission, and in adaptive/protective mechanisms of neuronal responses to hypoxia. However, the putative role of miRNA-132/212 in the response of synaptic transmission to ischemia remained unexplored. Using hippocampal slices from female miRNA-132/212 double-knockout mice in an established electrophysiological model of ischemia, we here describe that miRNA-132/212 gene-deletion aggravated the deleterious effect of repeated oxygen-glucose deprivation insults on synaptic transmission in the dentate gyrus, a brain region crucial for learning and memory functions. We also examined the effect of miRNA-132/212 gene-deletion on the expression of key mediators in cholinergic signaling that are implicated in both adaptive responses to ischemia and hippocampal neural signaling. miRNA-132/212 gene-deletion significantly altered hippocampal AChE and mAChR-M1, but not α7-nAChR or MeCP2 expression. The effects of miRNA-132/212 gene-deletion on hippocampal synaptic transmission and levels of cholinergic-signaling elements suggest the existence of a miRNA-132/212-dependent adaptive mechanism safeguarding the functional integrity of synaptic functions in the acute phase of cerebral ischemia.

## 1. Introduction

Oxygen is a key determinant of mammalian life and severe oxygen deprivation is most often its antithesis. Oxygen deprivation accompanied by a sudden disruption of substrate delivery to brain tissue, as occurring in the form of ischemic stroke, triggers a devastating cascade of pathophysiological events, culminating in irreversible dysfunction and loss of CNS tissue [1]. The resulting loss of neural function in stroke patients constitutes a leading cause of disability worldwide [2], as the armamentarium of pharmacological interventions is still slim and the temporal window for effective drug treatment is narrow [3,4]. Therefore, advances in the understanding of the molecular and functional mechanisms engaged in brain responses to oxygen glucose deprivation (OGD) are of paramount importance.

In an effort to illuminate pathophysiological hallmarks of ischemic brain injury and ultimately establish novel therapeutic targets, micro-RNAs (miRNAs) have entered the spotlight of scientific inquiry (e.g., see [5,6,7,8,9] for a review). Micro-RNAs, a class of single stranded (∼21–22 nucleotide long) non-coding RNAs, are highly evolutionarily conserved, potent and selective post-transcriptional regulators of mRNA translation [10,11,12,13]. In the central nervous system (CNS), miRNAs have been implicated in diverse physiological processes such as neuronal development [14,15,16,17], adult neurogenesis [18], and synaptic transmission and plasticity [19,20,21,22,23]. Correspondingly, dysregulation of miRNAs has been associated with hallmarks of pathogenesis for a variety of debilitating neurological disorders including epilepsy [24,25,26,27], Alzheimer’s disease [28,29,30,31], and ischemic brain injuries [5,6,7,8,9]. Indeed, rising evidence on the differential regulation of miRNAs in the course of cerebral ischemia-reperfusion injuries has led to a vivid debate on their mechanistic functions in the pathophysiology of ischemia–reperfusion insults and their putative role as biomarkers, and detrimental or neuroprotective agents [5,6,7,8,9,32].

Interestingly, the neuron-enriched miRNA 132/212 cluster has been implicated both in the physiological regulation of the molecular mechanisms underlying higher order cognitive functions such as learning and memory ex and in vivo [16,21,22,33], and in resilience to hypoxic and ischemic insults of various cell lines in vitro [34,35,36]. However, miRNA 132/212 regulation in cerebral ischemia–reperfusion injuries and its putative role in the pathophysiology of ischemic injury still remains controversial [35,36,37]. Incidentally, one of the brain regions most prominently associated with learning and memory, the hippocampal formation, is also one of the most vulnerable to hypoxic and ischemic insults [38,39,40]. From a clinical perspective, these overlaps might merit further investigation, as post-ischemic cognitive impairments are particularly prevalent, debilitating and difficult to treat [41,42], even after successful initial recovery [43] or comparatively mild cerebral ischemia [44].

Previous research has demonstrated that the in vivo overexpression of miRNA-132 using intracerebroventricular application of miRNA-132 mimics, starting 24 h after Middle Cerebral Artery Occlusion (MCAO), ameliorated ischemic tissue damage and fostered functional recovery, while the application of miRNA-132 inhibitors reversed mitigating effects of electroacupuncture (EA) on stroke [45]. Collectively, previous data highlighted an antiapoptotic and neuroprotective role of the miRNA-132/212 cluster in the cellular response to OGD in vitro [35,36], as well as a role in neuroprotection and post-ischemic regeneration in vitro and in vivo [45]. A putative role of the miRNA 132/212 cluster in the regulation of synaptic transmission during the acute phase of OGD has yet to be elucidated. Although independently implicated as a key regulator of synaptic transmission [21,22] and the adaptive response to ischemia [35,36], the putative role of the miRNA 132/212 cluster in the acute response of synaptic transmission to ischemia–reperfusion insults remains scarcely researched. Thus, we adapted a widely established ex vivo model of OGD (see [46] for a methodological review) to study the role of miRNAs 132/212 in the response of synaptic transmission to repeated ischemia–reperfusion insults in the dentate gyrus, a brain structure critical for learning and memory, and adult neurogenesis [47,48]. Furthermore, we conducted biochemical analyses of miRNA 132/212 targets, including cholinergic receptors known to be implicated in synaptic transmission and plasticity, as well as in the neuronal response to OGD. Our results lay out, to the best of our knowledge, the first electrophysiological and biochemical characterization of the influence of miRNA-132/212 gene deletion on synaptic transmission under OGD in a mammalian hippocampal circuit.

## 2. Materials and Methods

### 2.1. Animals

Wild-type (WT) C57Bl/6N and transgenic miRNAs 132/212 KO (miRNA 132/212^−/−^) adult female mice bred on a C57Bl/6 background (15–20 weeks old) were analyzed in this study. As female subjects are underrepresented in biomedical research in general and in neuroscience in particular [49], a bias that expands to clinical trials in human populations [50], we chose to research female mice. All the transgenic mice used here were verified by Genotyping PCR and stem from the line generated and characterized by Remenyi et al. [21]; their littermates, all with verified WT genotype, served as controls. All animals were housed in standard Plexiglas housing cages (Tecniplast SAS, Zona Industriale Novoleto, 54027 PONTREMOLI (MS), Italy) in groups of three to five mice per cage, with access to food and water ad libitum. The colony room was kept at controlled temperature (22 ± 2 °C) and lighting (200 ± 20 lx) conditions, with an automatically controlled 12 h light/dark cycle (Hager EH210, Hager Electro Ges.m.b.H.; Vienna, Austria) with light periods starting at 7:00 AM. All experimental procedures followed Bundesministerium für Wissenschaft und Forschung of Austria directives (BMWF-66.009/0200-WF/V/3b/2016). All procedures were conducted in accordance with the ARRIVE guidelines and the U.K. Animals Scientific Procedures Act (Scientific Procedures Act, 1986, and associated guidelines, EU Directive 2010/63/EU for animal experiments).

### 2.2. Electrophysiology

#### 2.2.1. Hippocampal Slices Preparation

Acutely dissected hippocampal slices obtained from miRNA 132/212^−/−^ mice and WT littermates (*n* = 6–7 animals per experimental group) were prepared as previously described [22,51,52,53], with slight modifications. Briefly, mice were sacrificed by swift cervical dislocation and subsequent sharp-blade decapitation. Utilizing one midline incision from the foramen magnum to the frontal suture and two basolateral incisions from the foramen magnum outwards, the parietal bones were mobilized and removed. The brains were removed gently with a spatula and immediately submerged in an ice-cold aCSF solution of the following composition (in mM): 125 NaCl, 2.5 KCl, 20 NaHCO_3_, 2.5 CaCl_2_, 1 MgCl_2_, 25 D-glucose and 1 NaH_2_PO_4_ (pH 7.35–7.40). Coronal hippocampal slices (400 μm nominal thickness) were cut while submerged in ice-cold, carbogenated aCSF (95% O_2_/5% CO_2_), using a vibrating microtome (Vibratome 7000smz-2, Campden Instruments Ltd.; PO Box 8148, Loughborough, Leics.; LE12 7TJ. U.K.) at a frequency of 90 Hz, an amplitude of 0.75 mm and speed of 0.12 mm/s. Cutting was followed by separation of hemispheres and immediate transfer to a custom-build recovery chamber filled with carbogenated aCSF at 32 °C. Slices were allowed to recover for a minimum of 1.5 h before electrophysiological recording.

#### 2.2.2. Extracellular Recordings

**Figure 1:**
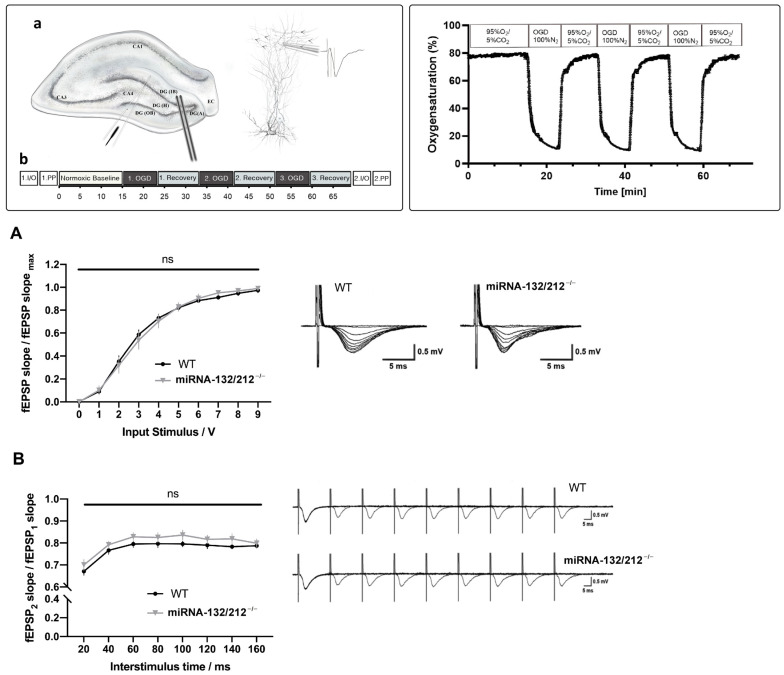
miRNA-132/212 deletion did not alter basal synaptic transmission and paired-pulse inhibition (PPI) in the hippocampal dentate gyrus. Inset (1) Schematic representation of the electrophysiological recording setting. (**a**) Diagram of a hippocampal slice, with a schematic representation of the histological layering of the dentate gyrus (middle inset). The lower left inset depicts the positioning of the stimulating and recording electrode. The right inset illustrates the positioning of the recording electrode in relationship to the dendritic layer of the neuronal granule cells and a representation of a recorded field excitatory postsynaptic potential (fEPSP). Arrows denote the flow of inputs from the perforant pathway. (**b**) Schematic synthesis of the experimental protocol for electrophysiological recordings. CA 1–4: Cornu Ammonis areas 1–4, DG: Dentate gyrus, (A): Apex, (H): Hilus, (IB): Inner Blade, (OB): Outer Blade, EC: Entorhinal cortex, fEPSP: field excitatory postsynaptic potential, I/O: Input Output, PP: Paired pulse stimulation, OGD: Oxygen Glucose Deprivation. Inset (2) Temporal course of the oxygen saturation measured in the recording chamber during repeated episodes of OGD. Superfusion of the recording chamber with 100% N2 gassed OGD aCSF led to a sharp drop of oxygen saturation below 20% within 3 min bottoming at less than 12% oxygen saturation at the end of the OGD period. Reperfusion with carbogenated aCSF (95%O_2_/5%CO_2_) increased the oxygen saturation back to values greater than 60% oxygen saturation in less than 1 min. Graph derived from optode recorded oxygen saturation data of three independent recording days. aCSF: artificial cerebrospinal fluid, OGD: Oxygen Glucose Deprivation. (**A**) I/O curves generated by plotting normalized fEPSP slope changes versus increasing pulses of input stimulation voltages obtained in WT (*n* = 7) and miRNA-132/212^−/−^mice (*n* = 6) hippocampal slices. Two-way repeated measures ANOVA did not reveal statistically significant differences between genotypes before the implementation of the OGD treatment. Right inset: Representative raw fEPSP traces recorded from hippocampal slices obtained from WT mice (left) and miRNA-132/212^−/−^mice (right) in response to increasing stimulus intensities show no apparent differences. (**B**) PPI ratios (second pulse/first pulse) plotted against inter-pulse intervals from 20 to 160 ms. No statistically significant differences (“ns” in the figure) between genotypes were detected by two-way repeated measures ANOVA. Right inset: Depiction of representative raw fEPSP traces obtained during the PPI protocol described in the materials and methods section in the dentate gyrus of WT (upper trace) and miRNA-132/212^−/−^ mice (lower trace), without macroscopic differences. Data is presented as mean ± SEM. fEPSP: field excitatory postsynaptic potential, I/O: Input Output, OGD: Oxygen Glucose Deprivation, PPI: Paired Pulse Inhibition.

Slices were transferred to a customized low volume (2 mL) submersion-type recording chamber perfused with carbogenated or nitrogenated aCSF solution at a constant flow rate of 4 mL/min. Slices were allowed to accommodate for at least 5 min before any further manipulation. Evoked field excitatory postsynaptic potentials (fEPSPs) were obtained using borosilicate glass micropipettes, using a pipette puller (Flaming/Brown Micropipette Puller Model P-87, Sutter Instrument, Novato, CA 94949, USA). Micropipette tips were backfilled with aCSF and yielded a tip resistance of (3 ± 1) MΩ. Teflon-coated tungsten wire bipolar stimulating electrodes (~50 μm diameter tip) connected to an ISO-STIM 01D isolator stimulator (npi electronic GmbH, Bauhofring 16, D-71732 Tamm, Germany) were used to evoke fEPSPs. An AxoClamp-2B amplifier, Digidata-1440 interface (Axon Instruments, Molecular Devices, 660-665 Eskdale Road, Winnersh Triangle, Wokingham, Berkshire RG41 5TS, UK) and pClamp-11 software (Molecular Devices, 660-665 Eskdale Road, Winnersh Triangle, Wokingham, Berkshire RG41 5TS, UK) were used for data acquisition and analysis. As per previously established protocols [53,54], stimulating and recording electrodes were located approximately 400–500 μm apart in the middle molecular cell layer of the dentate gyrus, in dorsal to mid-coronal slices, with the stimulating electrode placed at the medial perforant pathway (MPP) as depicted in Figure 1 (inset 1). fEPSPs were recorded at 0.1 Hz frequency and linear fit between 20% and 80% of the decaying phase of each fEPSP slope was used for further analysis. To generate standard Input/Output (I/O) curves, square pulses of increasing stimulus intensity (duration 200 μs, 10 s inter-pulse interval, 0–9 V with 1 V increments) were delivered at the medial perforant pathway (MPP) to elicit fEPSP responses. For I/O curves, fEPSP slopes were normalized to the highest inducible slope value, for each slice recording. The stimulus intensity for the assessment of basal synaptic transmission under the OGD treatment protocol and paired pulse recordings was adjusted to elicit 80% of the maximal inducible amplitude established with I/O curves, and kept constant for the rest of the recordings. Paired-pulse inhibition (PPI) fEPSP responses were evoked by applying two consecutive stimulation pulses, with increasing interstimulus intervals (8 pulses, 20–160 ms interstimulus intervals with 20 ms increments). The effects of repeated OGD treatment on synaptic transmission were normalized to the last 5 min of normoxic and euglycemic baseline recording, preceding the OGD treatment protocol and then analyzed by comparing fEPSP slopes during OGD and recovery. Data were averaged within 30 s intervals (3 consecutive sweeps), further averaged within animals, and compared between miRNA-132/212^−/−^ and WT mice. A total of *n* = 34 slices were included in the analysis. On average, *n* = 2–3 slices per animal and a total of *n* = 7 WT and 6 miRNA 132/212^−/−^ animals were analyzed.

#### 2.2.3. Oxygen Glucose Deprivation Protocol

Standard aCSF, of the following composition (in mM): 125 NaCl, 2.5 KCl, 20 NaHCO_3_, 2.5 CaCl_2_, 1 MgCl_2_, 25 D-glucose and 1 NaH_2_PO_4_, was modified as follows to achieve an OGD-aCSF. Glucose was replaced with equimolar sucrose and the solution was continuously gassed with 100% N_2_ gas instead of a 95% O_2_/5% CO_2_ gas mixture, as previously established [55]. pH was monitored and kept constant at 7.35–7.40 throughout the experiment by buffering the OGD-aCSF with 10 mM NaHCO_3_ and 25 mM HEPES. Firstly, evoked potentials were recorded for 15 min under euglycemic and normoxic conditions by superfusing the slices with standard aCSF, gassed with 95% O_2_/5% CO_2_, before any treatment. The slices were then superfused with OGD-aCSF (100% N_2_) for a total of three OGD 8 min intervals. Each OGD interval was followed by a recovery period of 10 min, in which the slices were superfused with standard, euglycemic, normoxic aCSF (95% O_2_/5% CO_2_).

After 15 min of baseline recording under normoxic conditions (95% O_2_/5% CO_2_), slices were superfused with OGD aCSF three times for 8 min with intervals of 10 min as recovery periods under normoxic and euglycemic aCSF superfusion (95% O_2_/5% CO_2_), following every OGD period (Figure 1 (inset 2)). Changes in solutions reached the recording chamber within 50 s, which was taken into account in our calculations. To estimate the local oxygen saturation of the aCSF in the recording chamber an optode (Needle type housing oxygen microsensor PSt7-02, tip diameter <50 μm, OXY-1 ST transmitter (PreSens -Precision Sensing GmbH, Am BioPark 11, 93053 Regensburg, Germany) was placed approximately 100–200 μm vertically above the recording site. Beforehand, we used a two-point calibration process as per the manufacturer’s instructions, with minor adjustments as previously established [56]. To achieve a nominally 0% oxygen solution (Cal 0) an 80 mM sodium sulfite solution (Na_2_SO_3_ in ddH_2_O) was prepared. Cobalt nitrate Co(NO_3_)_2_ standard solution (ρ(Co) = 1000 mg/L; concentration 0.5 mol/L in nitric acid) was used to catalyze oxygen desaturation. To estimate the maximal oxygen saturation of carbogenated aCSF (Cal 100), standard aCSF was bubbled with 95% O_2_/5% CO_2_ for at least 1 h before calibration. In accordance with previously published data [56], the maximal achievable PO_2_ of aCSF in our experimental setup was estimated to be (720 ± 10) Torr. Oxygen saturation is reported as percentage of the maximal O_2_ saturation of continuously carbogenated aCSF. Oxygen saturation measured in the recording chamber ranged from 78.450 ± 0.024% during normoxia to 10.380 ± 0.046% under OGD treatment. (Figure 1 (inset 2)) shows the local oxygen saturation in the recording chamber throughout the OGD treatment.

### 2.3. Immunoblotting

We extracted brains and isolated whole hippocampi from female miRNA 132/212^−/−^ and WT control mice (*n* = 5 animals per group) as previously described, with minor modifications [22,51,52,53]. Brains were quickly extracted, following swift cervical dislocation and sharp blade decapitation, and immersed in ice-cold artificial cerebrospinal fluid (aCSF) with pH balanced to 7.35–7.40. The cerebellum was removed and the hippocampus was dissected, sampled individually, snap-frozen in liquid nitrogen and asservated at −80 °C until further processing. Lysis buffer (50 mM Tris, 150 mM NaCl, 1% TritonX-100 and 5 mM EDTA) and 1:100 Protease and Phosphatase Inhibitor Cocktail (PIC; Fisher Scientific, Dresdner Str. 89, Vienna (1200), Austria) was used to homogenize brain tissue. Homogenized tissue was incubated at 4 °C on a tube rotator for 12 h. Next, lysates were centrifuged at 12,000× *g* for 5 min in a bench top centrifuge (Heraeus Fresco 17 R-134a, Fisher Scientific, Dresdner Str. 89, Vienna (1200), Austria) at 4 °C. After determination of sample protein concentrations using the BCA protein assay kit, as per manufacturer’s instructions (Pierce™ BCA Protein Assay Kit—Thermo Fisher Scientific; Fisher Scientific, Dresdner Str. 89, Vienna (1200), Austria), 25 μg of protein homgenate were loaded per lane in subsequent electrophoresis, performed on a 10% sodium dodecyl sulfate (SDS)-polyacrylamide gel, followed by transfer to poly (vinylidene difluoride) (PVDF) membranes (Immobilon P Transfer Membrane, Merck Millipore, Merck Group, Merck GmbH. Zimbagasse 5, 1140 Wien, Austria). PVDF membranes were blocked in 5% bovine serum albumin (BSA, A8022, Sigma-Aldrich Chemie GmbH, Eschenstraße 5, 82024 Taufkirchen, Germany) and washed 3 times for 5 min with TBST. Next, membranes were incubated overnight, at 4 °C in solutions with diluted primary antibodies: Anti- α7-nAChR (ab10096, 56 kDa, 1:1000 dilution in 3% BSA in TBST), Anti-Acetylcholinesterase (AChE) (ab183591, 68 kDa, 1:1000 dilution in 1% BSA in TBST), Anti-Muscarinic M1 Acetylcholine Receptor (mAChR M1) (M9808, 85 kDa, 1:250 dilution in 3% BSA in TBST), anti-MeCP2 (3456, 75 kDa, 1:1000 dilution in 3% BSA in TBST). Goat anti-rabbit served as secondary antibody (1:3000; Cat No. 7074S, Cell Signaling Technology Europe B.V. Zweigniederlassung Deutschland. Hanauer Landstrasse 291 B, 60314 Frankfurt am Main, Germany). Anti-GAPDH primary antibodies (Cat No. MA5-15738 Thermo Fisher Scientific, 37 kDa, 1:3000 dilution in 5% BSA in TBST), with Goat anti-mouse secondary antibodies (1:5000 dilution in 5% Milk in TBST), were used as loading controls. Electrochemical luminescence (ECL) reagent (1705061, Bio-Rad; Bio-Rad Laboratories GesmbH, Hummelgasse 88, 1130 Wien, Austria) was used for blot development and the FluorChem HD2 system (Alpha Innotech, Alpha Innotech Corporation, 2401 Merced St, San Leandro, CA 94577, USA) was used for protein visualization. Protein quantification and densitometric analysis was performed using the downloaded ImageJ software (Rasband, W.S.; ImageJ, U. S. National Institutes of Health, Bethesda, MD, USA, https://imagej.nih.gov/ij/, accessed on 20 October 2020).

### 2.4. Statistical Analysis

Statistical analyses were performed using GraphPad Prism Software, Version 8.0 (GraphPad Software, 2365 Northside Dr. Suite 560, San Diego, CA 92108, USA). Statistical analyses of more than two groups were conducted using analysis of variances (ANOVA), as appropriate. Specifically, we used two-way repeated measures ANOVA (or mixed model ANOVA) followed by Bonferroni’s multiple comparisons test to analyze normalized fEPSP slopes obtained from miRNA 132/212^−/−^ and WT slices over time. For statistical analyses of data strictly from within subject designs, we performed one-way ANOVAs with repeated measures and subsequent Bonferroni’s multiple comparisons tests, where appropriate. *p*-values and the number of samples used (*n*) are reported in the main text and represented in the figures (* *p* < 0.05, ** *p* < 0.01, *** *p* < 0.001, **** *p*< 0.0001). All data is expressed as mean ± standard error. The threshold of statistical significance (*α*) was set at 0.05 in all analyses.

## 3. Results

### 3.1. Dentate Gyri Basal Synaptic Transmission and Short-Term Plasticity Are Unaffected by miRNA 132/212 Gene Deletion

Before implementing the OGD treatment protocols we assessed whether miRNA 132/212^−/−^ and WT dentate gyri differed in their basal synaptic transmission properties. To this aim, we used standard Input/Output protocol (Materials and Methods). Recordings obtained from miRNA 132/212^−/−^ and WT hippocampal slices showed virtually indistinguishable synaptic responses (Figure 1A). Two-way repeated measures ANOVA showed a statistically significant main effect of input voltage (**** *p* < 0.0001, F_1.342, 16.10_ = 316.3), but no significant main effect of genotype (^ns^P = 0.9894, F_1, 12_ = 0.0001839) or input voltage × genotype interaction (^ns^P = 0.8830, F_9, 108_ = 0.4834). The dentate gyrus circuitry has the capacity to respond to fast repetitive stimulation via the perforant pathway with a decrease of neurotransmitter release, which can be assessed by delivering a succession of two pulses of increasing interstimulus intervals and quantifying the paired pulse inhibition (PPI) [57,58]. This conserved form of short-term plasticity has not only been associated with higher cognitive functions, such as learning and memory, but its impairment is associated with early pathophysiological hallmarks in icto- and epileptogenesis following convulsant stimulation [59]. PPI can be quantified as a paired pulse ratio (PPI-ratio) by dividing the value of the measured amplitude of the second fEPSP by the amplitude of the first fEPSP. Thus, lower PPI-ratio values indicate more pronounced inhibitory short term plasticity.

Congruent with our previously published data [22] we found no effect of miRNA 132/212 gene disruption on paired-pulse inhibition in the dentate gyrus, a priori to any OGD treatment (see Figure 1B). Two-way repeated measures ANOVA showed a significant effect of inter-pulse intervals (**** *p* < 0.0001, F_3.990, 43.89_ = 68.59), with no effect of genotype (^ns^P = 0.2458, F_1, 11_ = 1.503) and no significant inter-pulse interval x genotype interaction (^ns^P = 0.6879, F_7, 77_ = 0.6806).

### 3.2. Dentate Gyri Synaptic Depression Following OGD Is Aggravated in miRNA 132/212^−/−^ Mice

**Figure 2:**
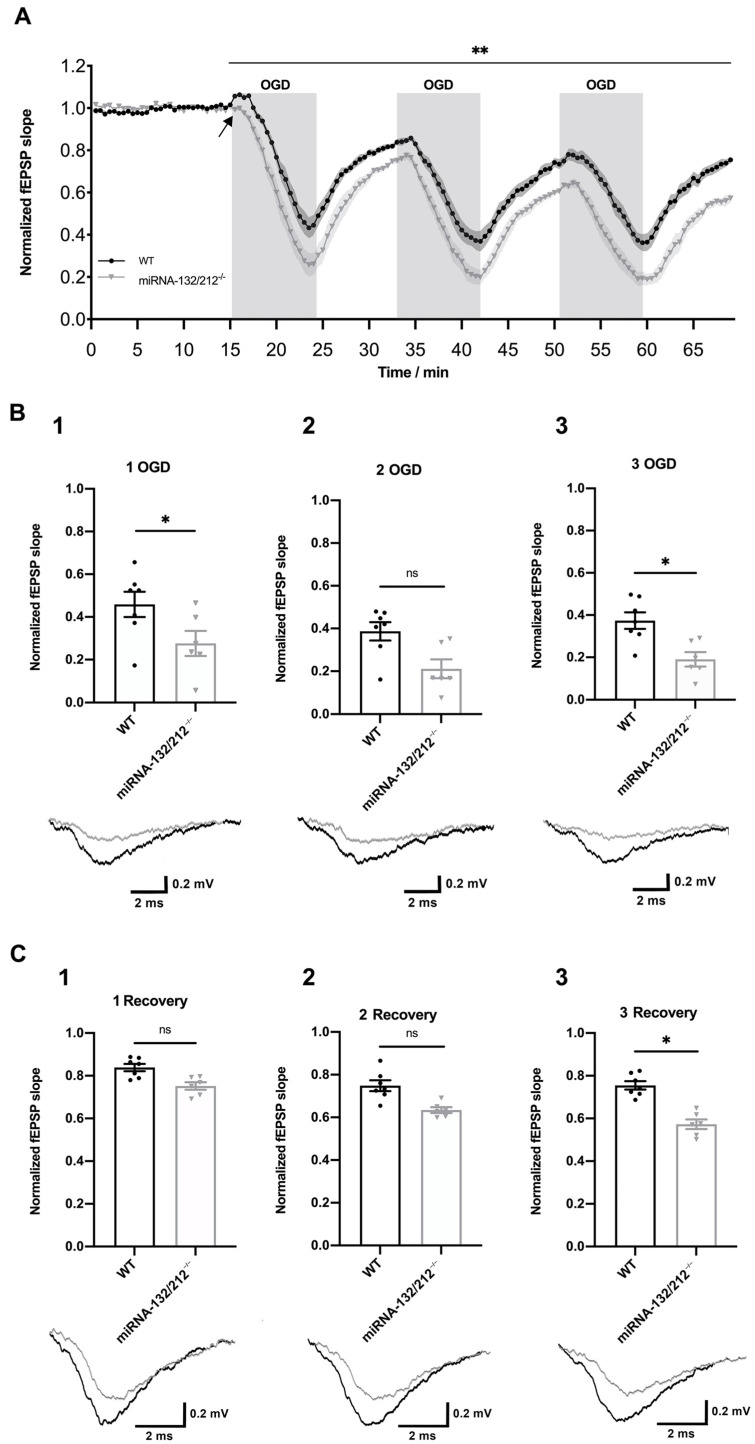
miRNA-132/212 gene disruption aggravated oxygen glucose deprivation (OGD) induced depression of synaptic transmission in the hippocampal dentate gyrus. (**A**) Temporal course of the fEPSPs slope changes elicited by three repeated 8 min long OGD episodes (100%N_2_, Glucose deprived aCSF), with interspersed 10 min recovery intervals (95%O_2_/5%CO_2_, 25 mM D-glucose aCSF), in slices obtained from WT (black) (*n* = 7) and miRNA-132/212^−/−^mice (gray) (*n* = 6). fEPSP slope values were normalized to the last 5 min of the initial baseline, under normoxic conditions. fEPSP slopes depressed and recovered to significantly lower values in slices obtained from miRNA-132/212^−/−^mice, as revealed by two-way repeated measures ANOVA. The black arrow denotes an initial potentiation upon the first OGD episode observed in WT slice recordings, which was virtually absent in recordings from miRNA 132/212^−/−^ slices. (**B**) Scatter plots depicting normalized fEPSP slopes recorded at the end of the first (**B1**), second (**B2**) and third (**B3**) OGD episodes. Bonferroni *post hoc* analysis revealed statistically significant genotype differences at the first and third but not second OGD interval. The insets below the scatter plots show representative fEPSP traces obtained from WT (black) and miRNA-132/212^−/−^mice (grey) at the corresponding time points. (**C**) Scatter plots depicting normalized fEPSP slopes recorded at the end of the first (**C1**), second (**C2**) and third (**C3**) recovery interval. Bonferroni *post hoc* analysis revealed statistically significant genotype differences at the end of the last, but not the preceding recovery intervals. The insets below the scatter plots depict representative fEPSP traces obtained from WT (black) and miRNA-132/212^−/−^mice (grey) at the corresponding time points. Data is presented as mean ± SEM. fEPSP: field excitatory postsynaptic potential, OGD: Oxygen Glucose Deprivation, ns: no statistical significance. *p* < 0.05 was considered significant. * *p* < 0.05, ** *p* < 0.01.

The superfusion of acutely dissected brain slices with OGD-aCSF has proven to be a useful, widely established ex vivo model of ischemia employed in the search for novel therapeutic targets to treat ischemia-induced brain injuries such as stroke e.g., [60,61,62,63,64,65], as well as for the illumination of the pathophysiological responses of neuronal circuits to ischemia e.g., [55,66,67,68,69,70]. Adapting a previously established approach [55], we used superfusion with OGD-aCSF to study the acute effects of repeated ischemia–reperfusion episodes on synaptic transmission in the dentate gyrus of acutely dissected slices obtained from miRNA 132/212^−/−^ mice and their respective WT littermates, who served as controls. We chose superfusion intervals with the OGD buffer for 8 min at a flow rate of 4 mL/min and a temperature of 32 °C, as in our pilot experiments this treatment robustly depressed synaptic transmission to ≤50% of its initial baseline, while allowing for recovery to 70–90% of the initial baseline within 10 min of normoxic/euglycemic reperfusion in WT slices. This protocol enabled us to study the effects of a series of multiple ischemia–reperfusion injuries on depression and recovery of synaptic transmission. We performed a two-way ANOVA analysis with repeated measures to compare synaptic transmission between the two genotypes over the course of the OGD protocol, which unveiled statistically significant main effects of time (**** *p* < 0.0001, F_137, 1507_ = 170.4) and genotype (** *p* = 0.0057, F_1, 11_ = 11.71) and a significant time x genotype interaction (**** *p* < 0.0001, F_137, 1507_ = 4.523) (see Figure 2A). Subsequent Bonferroni’s multiple comparisons tests for each 30 s interval revealed that fEPSP slopes recorded in miR132/212^−/−^ dentate gyri depressed to significantly lower values at the end of the first OGD episode (* *p* = 0.0442, t_1518_ = 3.606) and during the first minute of the first recovery in comparison to recordings in respective WT control slices (all * *p* < 0.05, (t_1518_)_min_ = 3.603; (t_1518_)_max_ = 3.924, t-values referring to lowest to highest value in this time frame) (see Figure 2B1). However, this difference did not remain statistically significant up to the end of the first recovery episode (^ns^P > 0.9999, t_1518_ = 1.707) (see Figure 2C1). Furthermore, the genotypes also did not differ significantly at the end of the second OGD (^ns^P = 0.0751, t_1518_ = 3.465) or the second recovery episode (^ns^P > 0.9999, t_1518_ = 2.258) (see Figure 2B2,C1,C2). However, the synaptic responses from slices obtained from miRNA 132/212^−/−^ and WT mice differed for almost the entire third OGD interval (all * *p* < 0.05, for every 30 s interval between 53.5 min and 59 min, t_1518_ = 3.603–4.710, t-values referring to lowest to highest value in this time frame), as well as the majority of the third recovery episode (all * *p*< 0.05, between 60.5 min and 64, 65, and 66 min, t_1518_ = 3.642–4.790, t values referring to lowest to highest t value in this time frame, respectively) with miRNA 132/212^−/−^ slices recovering to significantly lower fEPSP slope values at the end of the last recovery period, compared to the WT controls (* *p*= 0.0473, t_1518_ = 3.589) (see Figure 2A,B3,C3). Curiously, while slices obtained from WT mice robustly exhibited a small initial potentiation within the first 30 s of initial OGD, of on average of 9.441 ± 0.036% with respect to their initial prehypoxic/euglycemic baseline, this phenomenon was virtually absent in recordings obtained from miRNA 132/212^−/−^ mice (average depression of 0.013 ± 0.070% with respect to their initial baseline within the first 30 s of first OGD), (see Figure 2A). Collectively these data indicate that the depressive effect of repeated OGD on synaptic transmission is more pronounced in the absence of the miRNA 132/212 cluster and this difference between the genotypes becomes most pronounced with successive OGD insults.

### 3.3. Episodic OGD Impairs the Recovery of Dentate Gyri Synaptic Transmission in miRNA 132/212^−/−^

**Figure 3:**
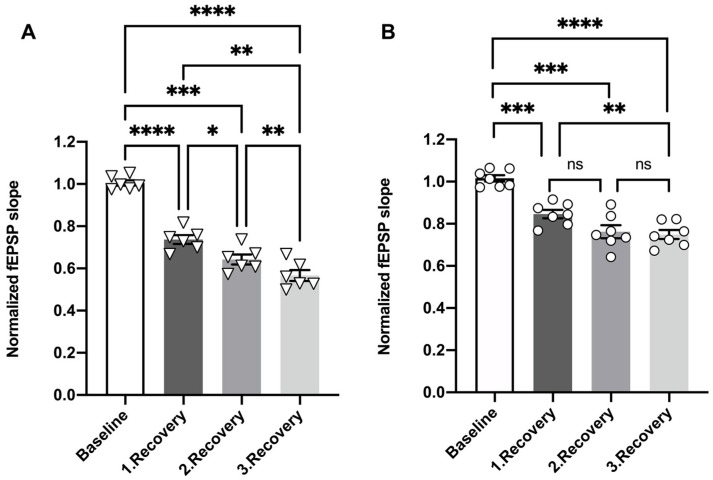
Repeated oxygen glucose deprivation (OGD) resulted in a steady impairment of synaptic transmission recovery in miRNA 132/212^−/−^ but not WT dentate gyri. Scatter plots depicting normalized fEPSP slope values at the normoxic, euglycemic baseline and the ends of the three recovery periods. (**A**) As revealed by within-subject analysis, fEPSP slopes recorded in slices obtained from miRNA 132/212^−/−^ mice (white filled inverted triangles) recovered to statistically significant lower values after OGD in comparison to each preceding recovery interval. (**B**) In contrast, in slices obtained from WT mice (white filled circles) within-subject analysis revealed statistically significant differences between fEPSP slope values reached at the first vs. the third recovery period, while values at the first vs. second and second vs. third recovery periods no longer differed significantly. Data is presented as mean ± SEM. fEPSP: field excitatory postsynaptic potential, OGD: Oxygen Glucose Deprivation. *p* < 0.05 was considered significant. * *p* < 0.05, ** *p* < 0.01, *** *p* < 0.001, **** *p* < 0.0001. ns: no statistical significance.

In order to further investigate the pronounced differences in the recovery of synaptic transmission at the last ischemia–reperfusion interval (Figure 2), we analyzed the efficiency of recovery over the course of the three ischemia–reperfusion episodes within both respective groups, using a within-subject design. We thus performed one-way ANOVAs with repeated measures, as well as subsequent Bonferroni’s multiple comparisons tests, to compare normalized fEPSP slope baseline values to the endpoints of each recovery interval for recordings from miRNA 132/212^−/−^ and WT controls, respectively. Strikingly, fEPSP slopes obtained from miRNA 132/212^−/−^ slices recovered to significantly reduced values at the end of each ischemia–reperfusion episode, in comparison to preceding recovery intervals, indicative of a cumulative effect of repeated OGD periods on the depression of synaptic transmission (see Figure 3A). One-way repeated measures ANOVA revealed a significant main effect of time in slices from miRNA 132/212^−/−^ mice (**** *p*< 0.0001, F_1.701, 8.503_ = 182.9). Bonferroni’s multiple comparisons tests showed statistically significant differences between the fEPSP slope values at normoxic, euglycemic baseline in comparison to slope values at the end of each recovery interval (**** *p* < 0.0001, t_5_ = 17.88, 17.59, Baseline vs. first and third recovery, respectively and *** *p* = 0.0001, t_5_ = 15.37, Baseline vs. second recovery). Furthermore, *post hoc* tests indicated statistically significant differences between the first vs. second recovery (* *p* = 0.0352, t_5_ = 4.596), first vs. third recovery (** *p* = 0.0034, t_5_ = 7.776) and second vs. third recovery (** *p* = 0.0040, t_5_ = 7.512).

One-way ANOVA with repeated measures also revealed a statistically significant main effect of time on normalized fEPSP slope values in WT slices (**** *p* < 0.0001, F_1.688, 10.13_ = 81.83, with fEPSP slopes reaching lower values after all recoveries in comparison to the initial baseline (Bonferroni’s multiple comparisons tests, all *** *p* < 0.001, t_6_ = 11.51, 9.13, for Baseline fEPSP slope values vs. values at the end of the first and second recovery, respectively and **** *p* < 0.0001, t_6_ = 18.71 for Baseline vs. third recovery). fEPSP slope values recovered to significantly lower values at the end of the third vs. the first recovery interval (** *p* < 0.0017, t_6_ = 7.559). However, comparing the fEPSP slope values reached at the end of the first vs. the second (^ns^P = 0.0636, t_6_ = 3.658), as well as the second vs. the third (^ns^P > 0.9999, t_6_ = 0.7425) recovery intervals, no statistically significant difference was observed (see Figure 3B). In summary, these findings indicate initial reduction in followed by a stabilization of synaptic transmission in response to repeated oxygen glucose deprivation in WT dentate gyri, which could not be observed in the slices obtained from mice lacking miRNA 132/212.

### 3.4. miRNA 132/212 Gene Deletion Promotes the Vulnerability of Short-Term Synaptic Depression to Deleterious Effects of OGD

**Figure 4:**
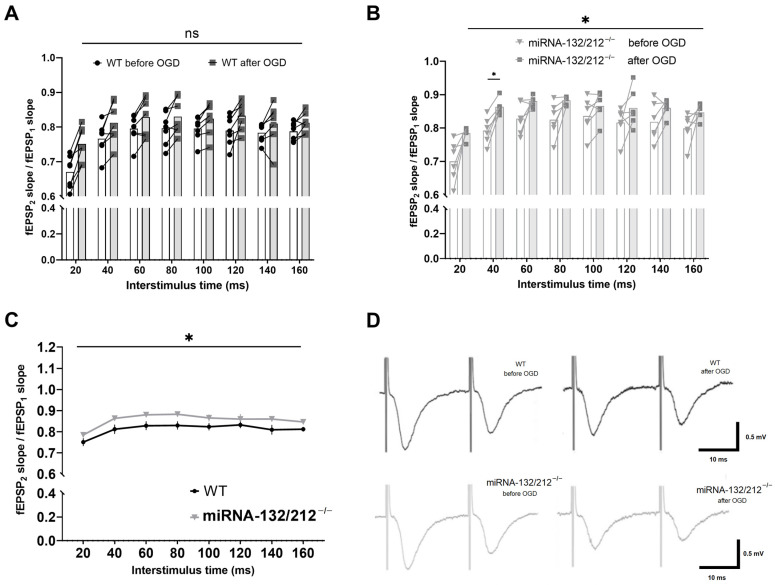
Repeated OGD impaired short term inhibitory plasticity in the absence of miRNA 132/212. Normalized and averaged PPI ratios (second pulse/first pulse) plotted against inter-pulse intervals from 20 to 160 ms, comparing responses from WT slices before and after the OGD treatment protocol: (**A**) PPI ratios, at ascending interstimulus intervals derived from WT slices and (**B**) miRNA-132/212^−/−^ slices before and after the OGD treatment protocol. (**C**) Comparison of PPI ratios, at ascending interstimulus intervals obtained from WT and miRNA-132/212^−/−^ slices after the OGD treatment. (**D**) Representative raw fEPSP traces obtained from WT (upper traces, black) and miRNA-132/212^−/−^ slices (lower traces, gray), before (left) and after (right) OGD treatment, applying a 20 ms inter-pulse interval paired pulse stimulation. Two-way repeated measures ANOVA revealed no statistically significant differences in the comparison of PPI ratios recorded in WT slices before and after OGD treatment (**A**). In contrast, PPI ratios increased significantly in miRNA-132/212^−/−^ slices after the OGD treatment, most pronounced at the 40 ms interstimulus interval (Bonferroni’s multiple comparisons tests) (**B**). Two-way repeated measures ANOVA revealed a significant difference between the genotypes after the OGD treatment (**C**). Data from *n* = 7 WT and *n* = 6 miRNA 132/212^−/−^ is presented as mean ± SEM. *p* < 0.05 was considered significant. * *p* < 0.05. ns: no statistical significance.

We subsequently examined whether OGD insults influence dentate gyrus short-term synaptic depression and whether this form of synaptic plasticity was differentially affected by OGD in miRNA 132/212^−/−^ and WT dentate gyri. We thus compared PPI ratios obtained before and after the OGD treatment in both genotypes using two-way repeated measures ANOVA. In slices obtained from WT mice, repeated measures ANOVA unveiled a predicted significant main effect of the interstimulus intervals (**** *p* < 0.0001, F_3.734, 44.81_ = 36.39), no statistically significant main effect of treatment (^ns^P = 0.0954, F_1, 12_ = 3.275) (see Figure 4A), and a statistically significant interstimulus interval x treatment interaction (* *p* = 0.0192, F_7, 84_ = 2.563).

In contrast, repeated measures ANOVA analyses of PPI ratios obtained from miRNA 132/212^−/−^ slices comparing PPI ratios from before and after OGD treatment yielded a significant main effect of inter-pulse interval (**** *p* < 0.0001, F_4.068, 40.68_ = 29.84), as well as significant main effect of treatment (before vs. after) (* *p* = 0.0276, F_1, 10_ = 6.634), with no statistically significant inter-pulse interval x treatment interaction (^ns^P = 0.1173, F_7, 70_ = 1.724). Bonferroni *post hoc* tests revealed a statistically significant increase in the PPI ratio after OGD in miRNA 132/212^−/−^ slices in response to pulses at 40 ms interpulse intervals (* *p* = 0.0415, t_7.916_ = 3.821). PPI ratios collected before and after OGD treatment from miRNA 132/212^−/−^ exhibited no statistically significant differences in response to paired pulse stimulation at any other inter-pulse interval (all ^ns^P ≥ 0.1360, all t ≤ 3.310) (see Figure 4B).

Repeated measures ANOVA comparing PPI ratios from both genotypes after OGD at different inter stimulus intervals revealed a statistically significant main effect of interstimulus interval (**** *p* < 0.0001, F_3.918, 43.09_ = 16.15) and a statistically significant main effect of genotype (* *p* = 0.0482, F_1, 11_ = 4.936), but no significant inter-pulse interval × genotype interaction (^ns^P = 0.8258, F_7, 77_ = 0.5081, Figure 4C).

These data show that the dentate gyri inhibitory short-term plasticity is significantly impaired by repeated OGD insults in the absence of the miRNA 132/212 cluster.

### 3.5. Basal Synaptic Transmission Remains Unaffected in the miRNA 132/212^−/−^ Hippocampus after Episodic OGD Insults

**Figure 5:**
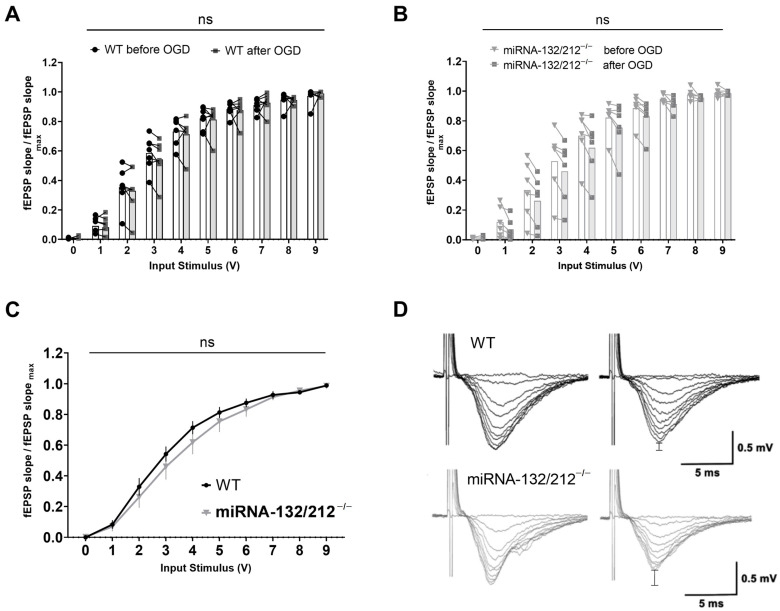
Repeated OGD did not significantly alter basal synaptic transmission in either genotype. I/O curves generated by plotting normalized fEPSP slope changes versus increasing pulses of input stimulation voltages obtained in WT (*n* = 7) and miRNA-132/212^−/−^ mice (*n* = 6) hippocampal slices. Separate two-way ANOVAs with repeated measures comparing basal synaptic transmission recorded before and after OGD treatment in WT dentate gyri (**A**) and miRNA-132/212^−/−^ dentate gyri (**B**) did not reveal statistically significant differences. (**C**) Normalized fEPSP slopes obtained from WT and miRNA-132/212^−/−^ slices after OGD treatment did not differ significantly. (**D**) Representative raw fEPSP traces recorded from hippocampal slices obtained from WT mice (upper row) and miRNA-132/212^−/−^ mice (lower row) in response to ascending stimulus intensities before (left) and after (right) OGD treatment. In both cases, the differences in the peak amplitudes before and after OGD are emphasized in the right panels by vertical bar indicators. Data is presented as mean ± SEM. fEPSP: field excitatory postsynaptic potential, OGD: Oxygen Glucose Deprivation. ns: no statistical significance.

Next, we sought to analyze the consequence of multiple ischemia–reperfusion insults on basal synaptic transmission responses to a range of stimulus intensities. We therefore compared standard I/O curves recorded before and after the OGD treatment protocol for both groups respectively, and compared I/O curves obtained from both genotypes after the OGD treatment using separate two-way ANOVAs with repeated measures. Comparing I/O curves obtained from WT slices before and after repeated OGD, we observed a statistically significant main effect of input voltage (**** *p* <0.0001, F_1.214, 14.57_ = 570.3), but no significant effect of treatment (before/after OGD treatment) (^ns^P = 0.8042, F_1, 12_ = 0.06426) and also no significant input-voltage x treatment interaction (^ns^P = 0.9607, F_9, 108_ = 0.3371, Figure 5A). Analyzing I/O curves from miRNA 132/212^−/−^ slices from before and after the OGD treatment repeated measures ANOVA revealed a significant main effect of input voltage (**** *p* < 0.0001, F_1.170, 11.70_ = 177.8), but not treatment (before vs. after OGD treatment) (^ns^P = 0.3928, F_1, 10_ = 0.7975), nor a significant input voltage x treatment interaction (^ns^P = 0.9758, F_9, 90_ = 0.2904) (see Figure 5B).

Comparing I/O curves obtained from WT and miRNA 132/212^−/−^ slices recorded after repeated OGD treatment, we observed a statistically significant main effect of input voltage (**** *p* <0.0001, F_1.156, 12.72_ = 301.0), but no significant main effect of genotype (^ns^P = 0.4247, F_1, 11_ = 0.6873), nor a significant input-voltage x genotype interaction (^ns^P = 0.6128, F_9, 99_ = 0.8048, Figure 5C).

In summary, the sensitivity of basal synaptic transmission in response to a range of different stimulus intensities remained unaffected after repeated OGD insults in either genotype.

### 3.6. miRNAs 132/212 Deletion Alters Protein Levels of AChE and mAChR-M1 in the Female Mouse Hippocampus

Abundant scientific literature has implicated miRNA 132/212 as a major regulator of key players in the cholinergic signaling of the brain [22,71,72,73] and the immune system [74,75,76].

Importantly, the confirmed miRNA 132/212 target Acetylcholinesterase (AChE) has been independently implicated in cognitive deficits in the aftermath of ischemic stroke [77]. Intriguingly, the expression of enzymes that regulate the availability of ACh at its site of action, such as AChE, have been shown to be involved in the regulation of the expression and function of nicotinergic and muscarinergic acetylcholine receptors [78,79]. Interestingly, the alpha-7 nicotinic receptor (nAChR-α7) and the muscarinic acetylcholine receptor M1 (mAChR-M1) have also been independently implicated in adaptive and maladaptive responses to hypoxia and ischemia [80,81,82,83,84,85]. Moreover, miRNA 132 has been shown to be involved in the regulation of methyl-CpG binding protein 2 (MeCP2) during ischemia preconditioning [37]. Furthermore, emerging evidence has recently started to unveil how crosstalk between the miRNA 132/212 cluster and cholinergic signaling influences synaptic transmission in the dentate gyrus [22], as well as cognitive correlates of synaptic communication in the hippocampus, such as learning and memory [33]. On the grounds of these observations and the above described aggravation of the depressive effect of repeated ischemia–reperfusion insults on synaptic transmission, we next explored the impact of miRNA 132/212 deletion on the expression of α7-nAChR, AChE, mAChR M1 and MeCP2 in the rodent hippocampus.

**Figure 6:**
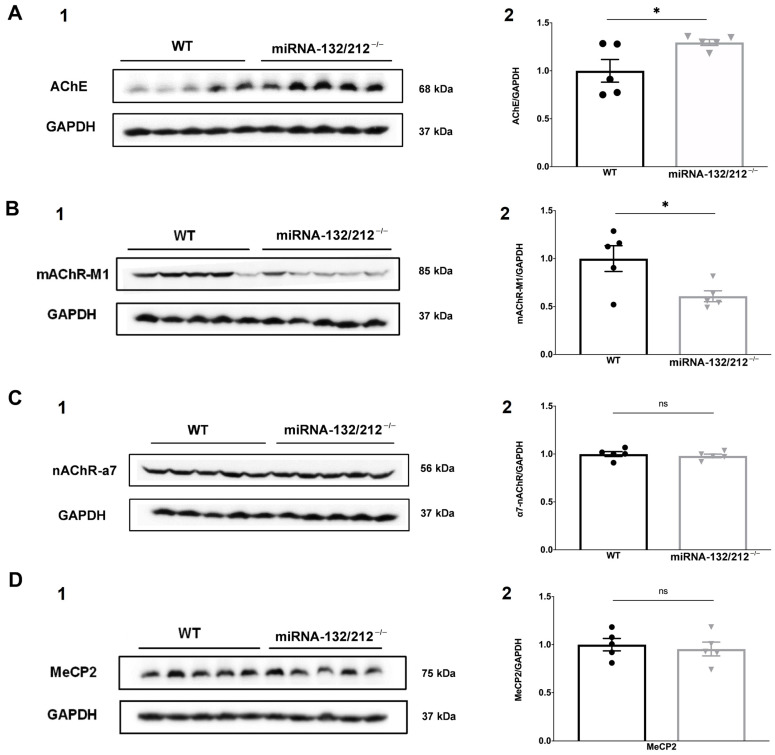
miRNA-132/212 deletion altered AChE and mAChR-M1 protein expression. Protein expression levels of AChE, mAChR-M1, nAChR-α7 and MeCP2 were determined by Western blot analysis from total cell lysates of female mouse bilateral hippocampi. The left columns (**A**–**D** and numbered with 1 in each case) depict representative immunoblots for the proteins of interest and GAPDH loading controls. The right columns (**A**–**D** and numbered with 2 in each case) depicts aligned scatter plots representing the protein levels of the respective proteins of interest, normalized to their respective GAPDH loading control protein levels, for lysates from miRNA 132/212^−/−^ and WT control mice. (**A**) Lysates from miRNA 132/212^−/−^ mice exhibited significantly higher AChE expression levels, as compared to their respective WT controls. (**B**) In contrast, lysates obtained from miRNA 132/212^−/−^ mice showed significantly lower mAChR-M1 expression levels, as compared to WT controls. However, samples from miRNA 132/212^−/−^ and WT control mice did not differ significantly regarding (**C**) nAChR-α7 and (**D**) MeCP2 protein expression. Data is expressed as mean ± SEM. *n* = 5 per group. AChE: Acetylcholinesterase, nAChR-α7: alpha-7 nicotinic receptor, mAChR-M1: muscarinic acetylcholine receptor M1, MeCP2: methyl-CpG binding protein 2. *p* < 0.05 was considered significant, * *p* < 0.05, ns: no statistical significance.

Cell lysates were derived from bilateral hippocampi collected from miRNA 132/212^−/−^ mice and their respective WT littermate controls and analysed using Western blots. Unpaired two-tailed Student’s *t*-test revealed significantly higher AChE protein expression in samples obtained from miRNA-132/212^−/−^ mice, as compared to WT controls (Figure 6A; * *p* = 0.0417; t_8_ = 2.422, *t*-test). On the other hand, lysates from miRNA-132/212^−/−^ mice exhibited significantly lower mAChR-M1 expression levels, in comparison to WT controls (Figure 6B; * *p* = 0.0279; t_8_ = 2.681, *t*-test), while no difference could be observed in the protein levels of nAChR-α7 (Figure 6C; ^ns^P = 0.5559; t_8_ = 0.6146, *t*-test), or MeCP2 (Figure 6D; ^ns^P = 0.6508; t_8_ = 0.4702, *t*-test).

## 4. Discussion

### 4.1. Modeling Ischemia Reperfusion Injuries Ex Vivo to Study the Effects of OGD on Synaptic Transmission

Studying the effects of OGD on synaptic transmission in brain slices ex vivo has proven to be a versatile approach in unveiling pathophysiological hallmarks of ischemia–reperfusion induced insults to neuronal circuits and identifying novel therapeutic targets, e.g., [46,55,61,62,63,67,68,86]. Although this methodology largely neglects the delicate interplay of all elements of the neurovascular unit in its broader in vivo context [87], it is particularly suited to study the acute functional responses of the isolated, intact neuronal circuitry to ischemia reperfusion insults, with high spatial and temporal resolution, in a controlled experimental setting. By adapting this widely established approach we here provided the first electrophysiological characterization of an involvement of the miRNA 132/212 cluster in the acute response of the dentate gyrus neuronal circuitry to repeated ischemia reperfusion insults. We showed that miRNA 132/212 deletion aggravates ischemia-induced depression of synaptic transmission in the mammalian dentate gyrus. These observations expand on the recent pioneering studies which demonstrated a protective role of miRNA 132 expression in the context of OGD in several cell lines [34,35,36]. Indeed, miRNA 132 overexpression has been shown to elicit antiapoptotic and neuroprotective effects in ischemia-challenged hippocampal neurons [35,36]. Moreover, miRNA 132 has been shown to be upregulated after OGD challenges [36], as well as in the course of ischemia preconditioning in isolated hippocampal neurons, which provided a protective effect in a subsequent OGD episode [35]. Here we explored how synaptic transmission in the dentate gyrus responds to a rapid succession of ischemia-reperfusion insults.

We here observed that in the absence of miRNA 132/212 the depression of synaptic transmission in the dentate gyrus, elicited by repeated OGD, is aggravated and the recovery from these insults is impaired. Interestingly, we observed a steady decrease in the efficiency of the synaptic transmission recovery from OGD induced depression in the absence of miRNA 132/212, as compared to WT controls. We thus propose the existence of a miRNA 132/212 dependent rapid adaptive response to ischemia–reperfusion insults, which might be involved in safeguarding the functional integrity of synaptic transmission in the dentate gyrus from ischemia-induced disruption.

While many studies have examined the influence of OGD on synaptic transmission in the hippocampal CA1 region, e.g., [55,60,61,62,63,64,66,70,88,89,90], the dentate gyrus has been studied less extensively in this paradigm, arguably because it has been shown to respond more resiliently to ischemia [86,91,92,93], making the study of ischemia–reperfusion induced damage towards it functional integrity more tedious. Nevertheless, several properties of the dentate gyrus merit further exploration of the mechanisms and consequences of ischemic insults to this region. The dentate gyrus has been conceptualized to serve a “gatekeeper” role in filtering cortical excitatory inputs to the hippocampus proper and its failure to effectively limit high frequency activity of excitatory inputs was associated with ictogenesis [94]. Additionally, we observed that repeated OGD insults significantly compromise the inhibitory short-term plasticity response of the dentate gyrus to high frequency paired pulse stimulation in the absence of the miRNA 132/212 cluster, particularly in the range of short inter-pulse intervals (40 ms). It is of note that we have not studied PPI in response to paired stimuli delivered at intervals longer than 160 ms, to which the observed effects might not necessarily extrapolate. Interestingly, the impairment of PPI in the dentate gyrus molecular layer has been observed to be an early consequence of seizure inducing perforant–path stimulation ex and in vivo and hence has been discussed as a pathological hallmark in icto- and epileptogenesis [59,95]. It has been widely established that repeated episodes of ischemia increase neuronal excitability and the occurrence of epileptiform discharges in the hippocampus [63,88,89,96]. Moreover, hypoxia-induced seizures have been linked to a lasting increase in dentate gyrus granule cell excitability [97]. Therefore, it appears that the delicate homeostasis of neuronal excitability in the hippocampal formation is particularly vulnerable to hypoxia and ischemia. Interestingly, while we here observed an OGD induced compromise of dentate gyrus inhibitory short term plasticity in the absence of the miRNA 132/212 cluster, previous research has in contrast indicated that miRNA 132 upregulation aggravates epileptiform discharges in an in vitro low Mg^2+^ neuronal culture model [26]. Moreover, the silencing of miRNA 132 decreased spontaneous recurrent seizures in an in vivo rodent model of lithium–pilocarpine induced temporal lobe epilepsy [24]. The contribution of miRNA 132/212 mediated signaling on neural excitability might thus depend highly on the precise context of the icto- and epileptogenic insult and differ across different neural circuits. Our findings thus invite further research on the putative role of the disruption of short term plasticity in the dentate gyrus in post ischemic icto- and epileptogenesis and a putative role of miRNA 132/212 mediated regulation of neural excitability, a topic of clinical relevance in light of the burden of early post stroke seizures [98].

### 4.2. The miRNA 132/212 Cluster: A Putative Link between Cholinergic Signaling, Hippocampal Synaptic Transmissions and the Resilience to Ischemia Induced Functional Impairment

Previous in vitro studies have implicated a neuroprotective and antiapoptotic role of miRNA 132 through the regulation of FOXO3 expression up to 48 h after an initial severe OGD insult [36]. Moreover, Zhao et al. (2018) established a SOX2 supression mediated beneficial effect of miRNA-132 upregulation on axonal regeneration after MCAO, by combining diverse in vivo and in vitro methodologies [45].

Here, we focused on the previously unexplored role of miRNA 132/212 signaling in the immediate response of synaptic transmission to transient OGD in the intact dentate gyrus circuitry. Given the emerging role of the miRNA 132/212 cluster as a key regulator of cholinergic signaling in the nervous [22,71,73,77] and other organ systems [72,74,75,76], we focused on how miRNA 132/212 disruption alters key mediators in cholinergic signaling, heavily implicated in the resilience to ischemic injuries. We here showed that the deletion of miRNA 132/212 results in a marked upregulation of AChE and a seemingly inverse downregulation of mAChR-M1 in female rodent hippocampi. AChE inactivates synaptic ACh signaling by rapid hydrolysis, discontinuing cholinergic transmission [99]. The herein observed combined upregulation of AChE together with mAChR-M1 downregulation bears the potential to critically blunt cholinergic signaling, the integrity of which appears crucial in the adaptive response to ischemia. Importantly, hypoxia has been shown to induce cognitive impairments and morphological damage in the rodent hippocampus in vivo (see also [100]), accompanied by increased AChE expression, while the administration of AChE inhibitors was able to alleviate hypoxia-induced cognitive impairments and neurodegeneration [101,102]. Moreover, recent data obtained from cerebrospinal fluid samples of human subjects not only demonstrated a robust negative regulatory relationship between miRNA 132 and its confirmed target AChE, but diminished miRNA 132 and heightened AChE levels were also associated with the development of post-stroke dementia [77]. Likewise, it has been shown that mAChR-M1 mediated signaling can be influenced by ischemic challenges, as well as consequences of ischemia in the central nervous system. Transient hypoxia has been shown to induce casein kinase 1 alpha (CK1α K46R)-dependent phosphorylation and sequestration of mAChR-M1 in vitro [103]. Correspondingly, mAChR-M1 mRNA was downregulated as a consequence of hypoxia in the rodent cortex and hippocampus in vivo [102]. Interestingly, mAChR-M1 mediated cholinergic signaling has been demonstrated to promote the accumulation and transcriptional activation of hypoxia inducible factor 1-alpha (HIF-1 α), a master regulator of the adaptive response to hypoxia in vitro [81] (see also [104]). This proposed role of mAChR-M1 mediated adaptation to hypoxia is underscored by the neuroprotective effect of hexahydropyrimidine derivatives, which were found to be putative mAChR-M1 ligands, in an in vivo rodent model of hypoxia [105].

### 4.3. Limitations and Perspectives

Although miRNA 132 and miRNA 212 share identical seed sequences, exhibit similar mature sequences and hence considerable overlap in their mRNA targets, the degree of functional redundancy of the mature miRNAs 132 and 212 in OGD and other physiological and pathophysiological contexts has not yet been fully elucidated [16,106]. Thus, although we here employed previously characterized total miR-132/212 double knockout mice [21], the data herein presented cannot provide conclusions on whether the observations reported are carried by the absence of either one of these miRNAs or their joint deletion. Our research therefore encourages further independent exploration of the individual contributions of both miRNA 132 and 212 to the early synaptic response to OGD. Moreover, we here employed a constitutive full-body knockout model in which this miRNA cluster of interest was absent during the entire ontogeny of the animal. Thus, it remains to be explored whether the observed aggravation of synaptic depression in response to OGD is due to a disruption of an acute, adaptive miRNA 132/212 dependent regulatory mechanism, or whether the chronic absence of the miRNA 132/212 cluster has primed the synaptic response prior to the insult.

Other authors [35] have also reported an absence of upregulation of miRNA 132 after 1 h following a presentation of 30 min of OGD as examined in primary dissociated neuronal cultures, whereas this miRNA was upregulated only 24 h after this OGD insult. Therefore, given that a timeframe of <2 h was used here for electrophysiological recordings as pertaining to the OGD protocol, we believe that the methodological limitations inherent to using acute hippocampal slices hamper the possibility to effectively examine the levels of miRNA 132 and miRNA 212 prior to and after OGD. Further research is therefore encouraged using alternative methods, including—for example—the use of organotypic slices or in vivo cerebral ischemia. Moreover, further in vivo experiments will be necessary to elucidate the possibility of an miRNA 132/212 dependent dynamic regulation of the expression of proteins involved in cholinergic signaling over the acute and subacute phases of OGD insults and its long term consequences.

Additionally, compared to the wide range of information available in the scientific literature, here we explored only a very narrow section of putative targets directly and indirectly implicated in miRNA 132/212 dependent signaling. Further research will be therefore necessary to advance towards a mechanistic understanding of how synaptic transmission is influenced by the miRNA 132/212 cluster in response to OGD. Moreover, while the OGD protocol used for this study was optimized to allow for the induction of a sequence of repeated OGD intervals, with intermittent recoveries, this protocol does not model the irreversible loss of synaptic transmission (as in e.g., [64]) and accompanying massive neuronal death as observed in the core of a ischemic lesion [107]. Whereas previous research has also utilized various cell culture-based assays to elaborate on the putative tissue-protective and antiapoptotic role of the miRNA 132/212 cluster in the neuronal response to severe OGD, in the time frame of 12 to 48 h [35,36] we here explored the role of this cluster in the acute response of synaptic transmission to a comparatively mild succession of brief OGD intervals. The long-term consequences of the reported observations, their relevance in the in vivo context of the intact neurovascular unit and whole brain circuitry, and their translational significance remain to be elucidated.

An additional and very important limitation of the work described here pertains to the potential physiological relevance of the sex of the subjects. Here we have exclusively focused on the study of female subjects and, consequently, the scope of our findings must be toned down accordingly to avoid generalizations. We have previously reported that α7-nAChR expression is upregulated in the absence of miRNA 132/212 in the hippocampi of male mice [22]. However, we did not observe an effect of miRNA 132/212 deletion on hippocampal α7-nAChR protein levels in female hippocampi. Given that sex-specific effects on the outcome of ischemic insults have recently been established for the effects of the miRNAs Let7f and mir363-3p [108], it may appear tempting to speculate on sex differences in miRNA 132/212 dependent regulations of cholinergic targets, and their putative role in the context of OGD. However, as our observations were derived from separate paradigms, any conclusions on putative sex-specific miRNA dependent regulations would be premature, as this research question merits a thorough independent investigation in its own right.

Moreover, previous research on the role of the miRNA 132/212 cluster in the modulation of central circadian rhythms unveiled highly strain- and light-regimen-specific effects of miRNA 132/212 deletion when comparing miRNA 132/212^−/−^ mice bred on C57BL/6N and 129/Sv backgrounds [109]. Independent replication of our findings in miRNA 132/212^−/−^ mice bread on different background strains might thus help to exclude the possibility of similar strain-specific roles of this cluster in the synaptic response to OGD.

Hippocampal synaptic plasticity events, including Long Term Potentiation (LTP) and Long Term Depression (LTD), have also been extensively investigated as potential mechanisms critical for the formation and regulation of learning and memory functions. We here observed that the miRNA 132/212 gene deletion promotes the vulnerability of short-term synaptic depression to repeated oxygen glucose deprivation. We, however, have not yet explored the susceptibility of LTP and LTD to OGD. As the miRNA 132/212 cluster has previously been shown by others (as well as by our group) to be involved in the regulation of hippocampal synaptic plasticity [21,22], the exploration of the putative involvement of the miRNA 132/212 cluster in the susceptibility of physiological synaptic plasticity to OGD as well as its possible involvement in aberrant forms of LTP (such as ischemic long term potentiation (iLTP) [110]) merits thus further investigations.

## 5. Final Conclusions

Here we described for the first time an involvement of the miRNA 132/212 cluster in the acute response of synaptic transmission to repeated ischemia reperfusion insults in the mammalian hippocampus, using an ex vivo electrophysiological approach. While previous research has implicated anti-apoptotic and regenerative effects of the miRNA 132/212 cluster via the silencing of FOXO3 [36] and SOX2 [45] in the hours and days following an OGD insult, our research focused on the early, acute functional effects of miRNA 132/212 dependent signaling on the integrity of synaptic transmission during repeated OGD events. Together with previous observations, focusing on OGD induced miRNA 132/212 regulation and tissue protection in the time frame of hours to days [35,36,45], our findings implicated diverse and pleiotropic miRNA 132/212 mediated adaptations to ischemic and hypoxic challenges.

This proposition might warrant further exploration of the manifold mechanisms regulated by miRNA 132/212 dependent signaling in the temporal course of ischemic challenges and their consequence on molecular signaling and cell viability.

We further explored the miRNA 132/212 dependent regulation of key mediators in the cholinergic signaling underlying both the physiological basis of higher order cognitive functions as well as the adaptive response to ischemia. We propose that the herein observed alterations in AChE and mAChR-M1 expression and aggravation of ischemia induced synaptic dysfunction in the dentate gyrus elicited by miRNA 132/212 deletion might help in the establishment of a link between miRNA 132/212, cholinergic signaling and ischemia associated cognitive impairment. Specifically, we speculate that the previously established link of concomitant miRNA 132 downregulation and AChE upregulation to post-ischemic dementia might be partially mediated by the failure of a putative miRNA 132 mediated mechanism to safeguard the functional integrity of hippocampal cholinergic synaptic signaling from ischemic disruption. Future research, addressing how miRNA 132/212 dependent signaling is involved in short- and long-term synaptic plasticity under OGD in vivo in various brain circuits over time, might help in the exploration of this research question. Our findings furthermore invite the elucidation of whether the expression of key players of cholinergic signaling such as ACh recpetors, or enzymes mediating the functional availability of ACh might be regulated by the miRNA 132/212 cluster over different phases during and after ischemic insults, in the in vivo context.

However, as the herein chosen paradigm can only illuminate a narrow aspect of miRNA 132/212 dependent signaling under OGD, any translational considerations must be phrased cautiously. For example, it has been recently shown that miRNA 132/212 overexpression is able to compromise blood–brain barrier integrity via regulation of tight junction properties in vitro [111], which might potentially aggravate ischemic injuries in vivo. Further independent research addressing the behavioral and brain morphological implications from our study are therefore encouraged. The current body of knowledge on miRNA 132/212 dependent signaling in the brain under hypoxia and ischemia needs thus to be further expanded to yield new diagnostic and therapeutic strategies in the treatment of ischemic brain injuries and their often devastating sequelae.

## Data Availability

All the data generated and/or analyzed in this study has been included in this article.

## References

[1] Campbell B.C., De Silva D.A., Macleod M.R., Coutts S.B., Schwamm L.H., Davis S.M., Donnan G.A. (2019). Ischaemic stroke. Nat. Rev. Dis. Primers.

[2] Johnson C.O., Nguyen M., Roth G.A., Nichols E., Alam T., Abate D., Abd-Allah F., Abdelalim A., Abraha H.N., Abu-Rmeileh N.M. (2019). Global, regional, and national burden of stroke, 1990–2016: A systematic analysis for the Global Burden of Disease Study 2016. Lancet Neurol..

[3] Powers W.J., Rabinstein A.A., Ackerson T., Adeoye O.M., Bambakidis N.C., Becker K., Biller J., Brown M., Demaerschalk B.M., Hoh B. (2019). Guidelines for the Early Management of Patients With Acute Ischemic Stroke: 2019 Update to the 2018 Guidelines for the Early Management of Acute Ischemic Stroke: A Guideline for Healthcare Professionals From the American Heart Association/American Stroke Association. Stroke.

[4] George P.M., Steinberg G.K. (2015). Novel Stroke Therapeutics: Unraveling Stroke Pathophysiology and Its Impact on Clinical Treatments. Neuron.

[5] Wu P., Zuo X., Ji A. (2012). Stroke-induced microRNAs: The potential therapeutic role for stroke. Exp. Ther. Med..

[6] Yang Y., Sandhu H.K., Zhi F., Hua F., Wu M., Xia Y. (2015). Effects of hypoxia and ischemia on microRNAs in the brain. Curr. Med. Chem..

[7] Yan H., Fang M., Liu X.Y. (2013). Role of microRNAs in stroke and poststroke depression. Sci. World J..

[8] Vasudeva K., Munshi A. (2020). miRNA dysregulation in ischaemic stroke: Focus on diagnosis, prognosis, therapeutic and protective biomarkers. Eur. J. Neurosci..

[9] Wang Y., Wang Y., Yang G.-Y. (2013). MicroRNAs in Cerebral Ischemia. Stroke Res. Treat..

[10] Ambros V. (2001). microRNAs: Tiny regulators with great potential. Cell.

[11] Bartel D.P. (2004). MicroRNAs: Genomics, biogenesis, mechanism, and function. Cell.

[12] Lai E.C. (2002). Micro RNAs are complementary to 3′ UTR sequence motifs that mediate negative post-transcriptional regulation. Nat. Genet..

[13] Zhang Y., Yun Z., Gong L., Qu H., Duan X., Jiang Y., Zhu H. (2018). Comparison of miRNA Evolution and Function in Plants and Animals. Microrna.

[14] Schaefer A., O’Carroll D., Tan C.L., Hillman D., Sugimori M., Llinas R., Greengard P. (2007). Cerebellar neurodegeneration in the absence of microRNAs. J. Exp. Med..

[15] Vo N., Klein M.E., Varlamova O., Keller D.M., Yamamoto T., Goodman R.H., Impey S. (2005). A cAMP-response element binding protein-induced microRNA regulates neuronal morphogenesis. Proc. Natl. Acad. Sci. USA.

[16] Wanet A., Tacheny A., Arnould T., Renard P. (2012). miR-212/132 expression and functions: Within and beyond the neuronal compartment. Nucleic Acids Res..

[17] Giraldez A.J., Cinalli R.M., Glasner M.E., Enright A.J., Thomson J.M., Baskerville S., Hammond S.M., Bartel D.P., Schier A.F. (2005). MicroRNAs regulate brain morphogenesis in zebrafish. Science.

[18] Cheng L.C., Pastrana E., Tavazoie M., Doetsch F. (2009). miR-124 regulates adult neurogenesis in the subventricular zone stem cell niche. Nat. Neurosci..

[19] Baby N., Alagappan N., Dheen S.T., Sajikumar S. (2020). MicroRNA-134-5p inhibition rescues long-term plasticity and synaptic tagging/capture in an Aβ(1-42)-induced model of Alzheimer’s disease. Aging Cell.

[20] Berentsen B., Patil S., Rønnestad K., Goff K.M., Pajak M., Simpson T.I., Wibrand K., Bramham C.R. (2020). MicroRNA-34a Acutely Regulates Synaptic Efficacy in the Adult Dentate Gyrus In Vivo. Mol Neurobiol..

[21] Remenyi J., van den Bosch M.W., Palygin O., Mistry R.B., McKenzie C., Macdonald A., Hutvagner G., Arthur J.S., Frenguelli B.G., Pankratov Y. (2013). miR-132/212 knockout mice reveal roles for these miRNAs in regulating cortical synaptic transmission and plasticity. PLoS ONE.

[22] Stojanovic T., Benes H., Awad A., Bormann D., Monje F.J. (2020). Nicotine abolishes memory-related synaptic strengthening and promotes synaptic depression in the neurogenic dentate gyrus of miR-132/212 knockout mice. Addict. Biol..

[23] Ye Y., Xu H., Su X., He X. (2016). Role of MicroRNA in Governing Synaptic Plasticity. Neural. Plast..

[24] Huang Y., Guo J., Wang Q., Chen Y. (2014). MicroRNA-132 silencing decreases the spontaneous recurrent seizures. Int. J. Clin. Exp. Med..

[25] Jimenez-Mateos E.M., Engel T., Merino-Serrais P., McKiernan R.C., Tanaka K., Mouri G., Sano T., O’Tuathaigh C., Waddington J.L., Prenter S. (2012). Silencing microRNA-134 produces neuroprotective and prolonged seizure-suppressive effects. Nat. Med..

[26] Xiang L., Ren Y., Cai H., Zhao W., Song Y. (2015). MicroRNA-132 aggravates epileptiform discharges via suppression of BDNF/TrkB signaling in cultured hippocampal neurons. Brain Res..

[27] Yuan J., Huang H., Zhou X., Liu X., Ou S., Xu T., Li R., Ma L., Chen Y. (2016). MicroRNA-132 Interact with p250GAP/Cdc42 Pathway in the Hippocampal Neuronal Culture Model of Acquired Epilepsy and Associated with Epileptogenesis Process. Neural Plast..

[28] El Fatimy R., Li S., Chen Z., Mushannen T., Gongala S., Wei Z., Balu D.T., Rabinovsky R., Cantlon A., Elkhal A. (2018). MicroRNA-132 provides neuroprotection for tauopathies via multiple signaling pathways. Acta Neuropathol..

[29] Herrera-Espejo S., Santos-Zorrozua B., Álvarez-González P., Lopez-Lopez E., Garcia-Orad Á. (2019). A Systematic Review of MicroRNA Expression as Biomarker of Late-Onset Alzheimer’s Disease. Mol. Neurobiol..

[30] Song Y., Hu M., Zhang J., Teng Z.Q., Chen C. (2019). A novel mechanism of synaptic and cognitive impairments mediated via microRNA-30b in Alzheimer’s disease. EBioMedicine.

[31] Salta E., De Strooper B. (2017). microRNA-132: A key noncoding RNA operating in the cellular phase of Alzheimer’s disease. FASEB J..

[32] Jimenez-Mateos E.M. (2015). Role of MicroRNAs in innate neuroprotection mechanisms due to preconditioning of the brain. Front. Neurosci..

[33] Hansen K.F., Sakamoto K., Aten S., Snider K.H., Loeser J., Hesse A.M., Page C.E., Pelz C., Arthur J.S., Impey S. (2016). Targeted deletion of miR-132/-212 impairs memory and alters the hippocampal transcriptome. Learn. Mem..

[34] Hong S., Lee J., Seo H.H., Lee C.Y., Yoo K.J., Kim S.M., Lee S., Hwang K.C., Choi E. (2015). Na(+)-Ca(2+) exchanger targeting miR-132 prevents apoptosis of cardiomyocytes under hypoxic condition by suppressing Ca(2+) overload. Biochem. Biophys. Res. Commun..

[35] Keasey M.P., Scott H.L., Bantounas I., Uney J.B., Kelly S. (2016). MiR-132 Is Upregulated by Ischemic Preconditioning of Cultured Hippocampal Neurons and Protects them from Subsequent OGD Toxicity. J. Mol. Neurosci..

[36] Sun Z.Z., Lv Z.Y., Tian W.J., Yang Y. (2017). MicroRNA-132 protects hippocampal neurons against oxygen-glucose deprivation-induced apoptosis. Int. J. Immunopathol. Pharmacol..

[37] Lusardi T.A., Farr C.D., Faulkner C.L., Pignataro G., Yang T., Lan J., Simon R.P., Saugstad J.A. (2010). Ischemic preconditioning regulates expression of microRNAs and a predicted target, MeCP2, in mouse cortex. J. Cereb. Blood Flow Metab..

[38] Schmidt-Kastner R., Freund T.F. (1991). Selective vulnerability of the hippocampus in brain ischemia. Neuroscience.

[39] Cervós-Navarro J., Diemer N.H. (1991). Selective vulnerability in brain hypoxia. Crit. Rev. Neurobiol..

[40] Kirino T., Tamura A., Sano K., Kogure K., Hossmann K.-A., Siesjö B.K., Welsh F.A. (1985). Selective Vulnerability of the Hippocampus to Ischemia—Reversible and Irreversible Types of Ischemic Cell Damage. Progress in Brain Research.

[41] Anderson C.A., Arciniegas D.B. (2010). Cognitive sequelae of hypoxic-ischemic brain injury: A review. NeuroRehabilitation.

[42] Sun J.H., Tan L., Yu J.T. (2014). Post-stroke cognitive impairment: Epidemiology, mechanisms and management. Ann. Transl. Med..

[43] Jokinen H., Melkas S., Ylikoski R., Pohjasvaara T., Kaste M., Erkinjuntti T., Hietanen M. (2015). Post-stroke cognitive impairment is common even after successful clinical recovery. Eur. J. Neurol..

[44] Jacquin A., Binquet C., Rouaud O., Graule-Petot A., Daubail B., Osseby G.V., Bonithon-Kopp C., Giroud M., Béjot Y. (2014). Post-stroke cognitive impairment: High prevalence and determining factors in a cohort of mild stroke. J. Alzheimers Dis..

[45] Zhao X., Bai F., Zhang E., Zhou D., Jiang T., Zhou H., Wang Q. (2018). Electroacupuncture Improves Neurobehavioral Function Through Targeting of SOX2-Mediated Axonal Regeneration by MicroRNA-132 After Ischemic Stroke. Front. Mol. Neurosci..

[46] Tasca C.I., Dal-Cim T., Cimarosti H. (2015). In vitro oxygen-glucose deprivation to study ischemic cell death. Methods Mol. Biol..

[47] Leuner B., Gould E. (2010). Structural plasticity and hippocampal function. Annu. Rev. Psychol..

[48] Liu H., Song N. (2016). Molecular Mechanism of Adult Neurogenesis and its Association with Human Brain Diseases. J. Cent. Nerv. Syst. Dis..

[49] Beery A.K., Zucker I. (2011). Sex bias in neuroscience and biomedical research. Neurosci. Biobehav. Rev..

[50] Cordonnier C., Sprigg N., Sandset E.C., Pavlovic A., Sunnerhagen K.S., Caso V., Christensen H. (2017). Women Initiative for Stroke in Europe (WISE) group. Stroke in women—from evidence to inequalities. Nat. Rev. Neurol..

[51] Cicvaric A., Bulat T., Bormann D., Yang J., Auer B., Milenkovic I., Cabatic M., Milicevic R., Monje F.J. (2018). Sustained consumption of cocoa-based dark chocolate enhances seizure-like events in the mouse hippocampus. Food Funct..

[52] Cicvaric A., Yang J., Bulat T., Zambon A., Dominguez-Rodriguez M., Kühn R., Sadowicz M.G., Siwert A., Egea J., Pollak D.D. (2018). Enhanced synaptic plasticity and spatial memory in female but not male FLRT2-haplodeficient mice. Sci. Rep..

[53] Cicvaric A., Yang J., Krieger S., Khan D., Kim E.J., Dominguez-Rodriguez M., Cabatic M., Molz B., Acevedo Aguilar J.P., Milicevic R. (2016). The brain-tumor related protein podoplanin regulates synaptic plasticity and hippocampus-dependent learning and memory. Ann. Med..

[54] Massa F., Koehl M., Wiesner T., Grosjean N., Revest J.M., Piazza P.V., Abrous D.N., Oliet S.H. (2011). Conditional reduction of adult neurogenesis impairs bidirectional hippocampal synaptic plasticity. Proc. Natl. Acad. Sci. USA.

[55] Furling D., Ghribi O., Lahsaini A., Mirault M.E., Massicotte G. (2000). Impairment of synaptic transmission by transient hypoxia in hippocampal slices: Improved recovery in glutathione peroxidase transgenic mice. Proc. Natl. Acad. Sci. USA.

[56] Hájos N., Ellender T.J., Zemankovics R., Mann E.O., Exley R., Cragg S.J., Freund T.F., Paulsen O. (2009). Maintaining network activity in submerged hippocampal slices: Importance of oxygen supply. Eur. J. Neurosci..

[57] Bekenstein J.W., Lothman E.W. (1991). Electrophysiological characterization of associational pathway terminating on dentate gyrus granule cells in the rat. Hippocampus.

[58] Bronzino J.D., Blaise J.H., Morgane P.J. (1997). The paired-pulse index: A measure of hippocampal dentate granule cell modulation. Ann. Biomed. Eng..

[59] Naylor D.E., Wasterlain C.G. (2005). GABA synapses and the rapid loss of inhibition to dentate gyrus granule cells after brief perforant-path stimulation. Epilepsia.

[60] Fusco I., Cherchi F., Catarzi D., Colotta V., Varano F., Pedata F., Pugliese A.M., Coppi E. (2019). Functional characterization of a novel adenosine A_2B_ receptor agonist on short-term plasticity and synaptic inhibition during oxygen and glucose deprivation in the rat CA1 hippocampus. Brain Res. Bull..

[61] Fusco I., Ugolini F., Lana D., Coppi E., Dettori I., Gaviano L., Nosi D., Cherchi F., Pedata F., Giovannini M.G. (2018). The Selective Antagonism of Adenosine A_2B_ Receptors Reduces the Synaptic Failure and Neuronal Death Induced by Oxygen and Glucose Deprivation in Rat CA1 Hippocampus in Vitro. Front. Pharmacol..

[62] Kim S.E., Ko I.G., Kim C.J., Chung J.Y., Yi J.W., Choi J.H., Jang M.S., Han J.H. (2016). Dexmedetomidine promotes the recovery of the field excitatory postsynaptic potentials (fEPSPs) in rat hippocampal slices exposed to oxygen-glucose deprivation. Neurosci. Lett..

[63] Levin S.G., Godukhin O.V. (2011). Anti-inflammatory cytokines, TGF-beta1 and IL-10, exert anti-hypoxic action and abolish posthypoxic hyperexcitability in hippocampal slice neurons: Comparative aspects. Exp. Neurol..

[64] Pugliese A.M., Traini C., Cipriani S., Gianfriddo M., Mello T., Giovannini M.G., Galli A., Pedata F. (2009). The adenosine A2A receptor antagonist ZM241385 enhances neuronal survival after oxygen-glucose deprivation in rat CA1 hippocampal slices. Br. J. Pharmacol..

[65] Sasaki R., Hirota K., Roth S.H., Yamazaki M. (2005). Anoxic depolarization of rat hippocampal slices is prevented by thiopental but not by propofol or isoflurane. Br. J. Anaesth..

[66] Fischer M., Reuter J., Gerich F.J., Hildebrandt B., Hägele S., Katschinski D., Müller M. (2009). Enhanced hypoxia susceptibility in hippocampal slices from a mouse model of rett syndrome. J. Neurophysiol..

[67] Pugliese A.M., Latini S., Corradetti R., Pedata F. (2003). Brief, repeated, oxygen-glucose deprivation episodes protect neurotransmission from a longer ischemic episode in the in vitro hippocampus: Role of adenosine receptors. Br. J. Pharmacol..

[68] Tanaka E., Yamamoto S., Kudo Y., Mihara S., Higashi H. (1997). Mechanisms underlying the rapid depolarization produced by deprivation of oxygen and glucose in rat hippocampal CA1 neurons in vitro. J. Neurophysiol..

[69] Wall A.M., Corcoran A.E., O’Halloran K.D., O’Connor J.J. (2014). Effects of prolyl-hydroxylase inhibition and chronic intermittent hypoxia on synaptic transmission and plasticity in the rat CA1 and dentate gyrus. Neurobiol. Dis..

[70] Youssef F.F., Addae J.I., McRae A., Stone T.W. (2001). Long-term potentiation protects rat hippocampal slices from the effects of acute hypoxia. Brain Res..

[71] Shaltiel G., Hanan M., Wolf Y., Barbash S., Kovalev E., Shoham S., Soreq H. (2013). Hippocampal microRNA-132 mediates stress-inducible cognitive deficits through its acetylcholinesterase target. Brain Struct. Funct..

[72] Hanieh H., Alzahrani A. (2013). MicroRNA-132 suppresses autoimmune encephalomyelitis by inducing cholinergic anti-inflammation: A new Ahr-based exploration. Eur. J. Immunol..

[73] Mishra N., Friedson L., Hanin G., Bekenstein U., Volovich M., Bennett E.R., Greenberg D.S., Soreq H. (2017). Antisense miR-132 blockade via the AChE-R splice variant mitigates cortical inflammation. Sci. Rep..

[74] Liu F., Li Y., Jiang R., Nie C., Zeng Z., Zhao N., Huang C., Shao Q., Ding C., Qing C. (2015). miR-132 inhibits lipopolysaccharide-induced inflammation in alveolar macrophages by the cholinergic anti-inflammatory pathway. Exp. Lung Res..

[75] Shaked I., Meerson A., Wolf Y., Avni R., Greenberg D., Gilboa-Geffen A., Soreq H. (2009). MicroRNA-132 potentiates cholinergic anti-inflammatory signaling by targeting acetylcholinesterase. Immunity.

[76] Wu M., Li N., Xu J., Wu L., Li M., Tong H., Wang F., Liu W., Feng Y. (2018). Experimental study on the regulation of the cholinergic pathway in renal macrophages by microRNA-132 to alleviate inflammatory response. Open Chem..

[77] Yang F.W., Wang H., Wang C., Chi G.N. (2020). Upregulation of acetylcholinesterase caused by downregulation of microRNA-132 is responsible for the development of dementia after ischemic stroke. J. Cell. Biochem..

[78] Bond C.E., Zimmermann M., Greenfield S.A. (2009). Upregulation of alpha7 Nicotinic Receptors by Acetylcholinesterase C-Terminal Peptides. PLoS ONE.

[79] Li B., Duysen E.G., Volpicelli-Daley L.A., Levey A.I., Lockridge O. (2003). Regulation of muscarinic acetylcholine receptor function in acetylcholinesterase knockout mice. Pharmacol. Biochem. Behav..

[80] Furukawa S., Sameshima H., Yang L., Ikenoue T. (2011). Acetylcholine receptor agonist reduces brain damage induced by hypoxia-ischemia in newborn rats. Reprod. Sci..

[81] Hirota K., Fukuda R., Takabuchi S., Kizaka-Kondoh S., Adachi T., Fukuda K., Semenza G.L. (2004). Induction of hypoxia-inducible factor 1 activity by muscarinic acetylcholine receptor signaling. J. Biol. Chem..

[82] Mou L., Gates A., Mosser V.A., Tobin A., Jackson D.A. (2006). Transient hypoxia induces sequestration of M1 and M2 muscarinic acetylcholine receptors. J. Neurochem..

[83] Sapozhnikova T.A., Borisevich S.S., Kireeva D.R., Gabdrakhmanova S.F., Khisamutdinova R.Y., Makara N.S., Gibadullina N.N., Khursan S.L., Zarudii F.S. (2019). Effects of novel hexahydropyrimidine derivatives as potential ligands of M1 muscarinic acetylcholine receptor on cognitive function, hypoxia-induced lethality, and oxidative stress in rodents. Behav. Brain Res..

[84] Shibata S., Koutaroh K., Tominaga K., Tanaka T., Watanabe S. (1992). Effect of muscarinic cholinergic drugs on ischemia-induced decreases in glucose uptake and CA1 field potentials in rat hippocampus slices. Eur. J. Pharmacol..

[85] Tohgi H., Utsugisawa K., Nagane Y. (2000). Protective effect of nicotine through nicotinic acetylcholine receptor α7 on hypoxia-induced membrane disintegration and DNA fragmentation of cultured PC12 cells. Neurosci. Lett..

[86] Maraula G., Traini C., Mello T., Coppi E., Galli A., Pedata F., Pugliese A.M. (2013). Effects of oxygen and glucose deprivation on synaptic transmission in rat dentate gyrus: Role of A2A adenosine receptors. Neuropharmacology.

[87] Iadecola C. (2017). The Neurovascular Unit Coming of Age: A Journey through Neurovascular Coupling in Health and Disease. Neuron.

[88] Doolette D.J., Kerr D.I.B. (1995). Hyperexcitability in CA1 of the rat hippocampal slice following hypoxia or adenosine. Brain Res..

[89] Godukhin O., Savin A., Kalemenev S., Levin S. (2002). Neuronal hyperexcitability induced by repeated brief episodes of hypoxia in rat hippocampal slices: Involvement of ionotropic glutamate receptors and L-type Ca(2+) channels. Neuropharmacology.

[90] Latini S., Bordoni F., Corradetti R., Pepeu G., Pedata F. (1999). Effect of A2A adenosine receptor stimulation and antagonism on synaptic depression induced by in vitro ischaemia in rat hippocampal slices. Br. J. Pharmacol..

[91] Aitken P.G., Schiff S.J. (1986). Selective neuronal vulnerability to hypoxia in vitro. Neurosci. Lett..

[92] Lalonde C.C., Mielke J.G. (2014). Selective vulnerability of hippocampal sub-fields to oxygen-glucose deprivation is a function of animal age. Brain Res..

[93] Shimizu H., Mizuguchi A., Aoki M. (1996). Differential responses between CA1 pyramidal cells and granule cells to ischemic insult in rat hippocampal slices. Neurosci. Lett..

[94] Dengler C.G., Coulter D.A. (2016). Normal and epilepsy-associated pathologic function of the dentate gyrus. Prog. Brain Res..

[95] Naylor D. (2002). Changes in nonlinear signal processing in rat hippocampus associated with loss of paired-pulse inhibition or epileptogenesis. Epilepsia.

[96] Schiff S.J., Somjen G.G. (1985). Hyperexcitability following moderate hypoxia in hippocampal tissue slices. Brain Res..

[97] Peng B.W., Justice J.A., He X.H., Sanchez R.M. (2013). Decreased A-currents in hippocampal dentate granule cells after seizure-inducing hypoxia in the immature rat. Epilepsia..

[98] Sarecka-Hujar B., Kopyta I. (2018). Poststroke epilepsy: Current perspectives on diagnosis and treatment. Neuropsychiatr. Dis. Treat..

[99] Soreq H., Seidman S. (2001). Acetylcholinesterase—New roles for an old actor. Nat. Rev. Neurosci..

[100] Kathner-Schaffert C., Karapetow L., Günther M., Rudolph M., Dahab M., Baum E., Lehmann T., Witte O.W., Redecker C., Schmeer C.W. (2019). Early Stroke Induces Long-Term Impairment of Adult Neurogenesis Accompanied by Hippocampal-Mediated Cognitive Decline. Cells.

[101] Muthuraju S., Maiti P., Solanki P., Sharma A.K., Amitabh, Singh S.B., Prasad D., Ilavazhagan G. (2009). Acetylcholinesterase inhibitors enhance cognitive functions in rats following hypobaric hypoxia. Behav. Brain Res..

[102] Muthuraju S., Maiti P., Pati S., Solanki P., Sharma A.K., Singh S.B., Prasad D., Ilavazhagan G. (2011). Role of cholinergic markers on memory function of rats exposed to hypobaric hypoxia. Eur. J. Pharmacol..

[103] Shirahata M., Hirasawa S., Okumura M., Mendoza J.A., Okumura A., Balbir A., Fitzgerald R.S. (2004). Identification of M1 and M2 muscarinic acetylcholine receptors in the cat carotid body chemosensory system. Neuroscience.

[104] Yang Y., Ju J., Deng M., Wang J., Liu H., Xiong L., Zhang J. (2017). Hypoxia Inducible Factor 1α Promotes Endogenous Adaptive Response in Rat Model of Chronic Cerebral Hypoperfusion. Int. J. Mol. Sci..

[105] Choudhary R., Malairaman U., Katyal A. (2017). Inhibition of 12/15 LOX ameliorates cognitive and cholinergic dysfunction in mouse model of hypobaric hypoxia via. attenuation of oxidative/nitrosative stress. Neuroscience.

[106] Aten S., Hansen K.F., Hoyt K.R., Obrietan K. (2016). The miR-132/212 locus: A complex regulator of neuronal plasticity, gene expression and cognition. RNA Dis..

[107] Xing C., Arai K., Lo E.H., Hommel M. (2012). Pathophysiologic cascades in ischemic stroke. Int. J. Stroke.

[108] Sohrabji F., Selvamani A. (2019). Sex differences in miRNA as therapies for ischemic stroke. Neurochem. Int..

[109] Kiessling S., Ucar A., Chowdhury K., Oster H., Eichele G. (2017). Genetic background-dependent effects of murine micro RNAs on circadian clock function. PLoS ONE.

[110] Lenz M., Vlachos A., Maggio N. (2015). Ischemic long-term-potentiation (iLTP): Perspectives to set the threshold of neural plasticity toward therapy. Neural. Regen. Res..

[111] Burek M., König A., Lang M., Fiedler J., Oerter S., Roewer N., Bohnert M., Thal S.C., Blecharz-Lang K.G., Woitzik J. (2019). Hypoxia-Induced MicroRNA-212/132 Alter Blood-Brain Barrier Integrity Through Inhibition of Tight Junction-Associated Proteins in Human and Mouse Brain Microvascular Endothelial Cells. Transl. Stroke Res..

